# Impact of Molar Furcations on Photodynamic Therapy Outcomes: A 6-Month Split-Mouth Randomized Clinical Trial

**DOI:** 10.3390/ijerph17114162

**Published:** 2020-06-11

**Authors:** Aymeric Courval, Laetitia Harmouche, Anne Mathieu, Catherine Petit, Olivier Huck, François Séverac, Jean-Luc Davideau

**Affiliations:** 1 Department of Periodontology, Dental Faculty, University of Strasbourg, 67000 Strasbourg, France; aycourval@gmail.com (A.C.); laetitia.harmouche@gmail.com (L.H.); am.anne.mathieu@gmail.com (A.M.); catherin.petit@gmail.com (C.P.); huck.olivier@gmail.com (O.H.); 2 Methodology and Biostatistics Group, Public Health Department, University Hospitals of Strasbourg, 67000 Strasbourg, France; francois.severac@chru-strasbourg.fr

**Keywords:** periodontal treatment effectiveness, non-surgical, photodynamic, molar furcation involvement, residual periodontal pocket

## Abstract

The effectiveness of adjunctive photodynamic treatment (PDT) to non-surgical periodontal therapy has been shown to depend on initial periodontal status. As molar furcation involvement impairs healing response to non-surgical periodontal therapy, the aim of this study was to evaluate the impact of furcation involvement on PDT outcomes. Thirty-six patients suffering from severe chronic periodontitis were included in a 6-month split-mouth randomized clinical trial. PDT applications used the toluidine blue O and a light-emitting diode (LED) with a red spectrum. Repeated PDT applications were performed in addition to non-surgical periodontal treatment at baseline and at 3-months. Pocket probing depth (PPD), plaque index, bleeding on probing, and clinical attachment level were recorded at baseline, and again at 3- and 6-months. Furcation sites of molars were compared to other sites of molars and non-molars. Multilevel analysis showed no PDT effect in molar furcation sites while an additional significant reduction (odds ratio = 0.67) of pockets with PPD > 5 mm in other sites at 3-months was measured. PPD reduction appeared delayed in molar furcation sites treated with PDT. There is no additional apparent benefit to use PDT in molar furcation sites for the reduction of pockets with PPD > 5 mm contrary to other sites.

## 1. Introduction

Periodontal diseases are infectious diseases and periodontal therapies aim to eliminate supra- and subgingival infections [1]. In addition to oral hygiene instructions, scaling and root planing (SRP) efficiently reduces periodontal pocket depth (PPD) and increases gain in the clinical attachment level [1]. However, SRP has some limitations, mainly in molars [2], essentially due to their complex anatomy [3]. In these multi-rooted teeth, furcation involvement has been shown to impair SRP response, limiting the reduction of PPD and clinical attachment loss (CAL), as well as increasing the risk of disease recurrence [4,5] and the persistence of PPD > 5 mm [6]. The complex morphology of furcation sites renders difficult the debridement of periodontal lesions and limits the removal of periodontal pathogens [3,7]. Accordingly, non-regenerative and regenerative surgical approaches have been proposed for the management of furcation involvement depending on furcation involvement severity [3,8]. However, long-term studies have shown that molar tooth loss is mainly and negatively influenced by the presence of furcation involvement during follow-up, with frequently associated odds ratios (OR) > 5 [9,10,11,12].

Due to this poor response to periodontal treatment at both short- and long-term, complementary antimicrobial treatments, such as locally delivered doxycycline [7,13], adjunctive photodynamic therapy (PDT) [14], and systemic antibiotic administration [15], have been proposed and evaluated. Indeed, systemic amoxicillin/metronidazole + SRP versus SRP alone improves PPD reduction and CAL at furcation sites but not furcation involvement degree [15]. Moreover, their frequent use could induce bacterial resistances [16] and significant adverse side effects [17]. Studies focusing on the specific effects of various local antimicrobial treatments of furcation involvement did not demonstrate any additional therapeutic values on PPD and furcation involvement changes compared to SRP alone. However no direct comparison of furcation site versus other site responses was performed [7,13,14].

Amongst local antimicrobial treatments, PDT is a non-invasive anti-infectious approach without any risk of side effects [18,19]. A photoactivatable agent (or photosensitizer) exposed to a light of compatible wavelength produces reactive oxygen radicals with antimicrobial properties [20,21,22]. Many studies have shown that the use of PDT during active periodontal treatment improved clinical treatment outcomes [23,24,25,26,27,28,29]. Among these outcomes, mean PPD, CAL reductions, and gingival inflammation have been mainly investigated [19]. However, the persistence of pockets with PPD > 5 mm after active treatment has been rarely studied [6,16,29,30,31]. Those residual pockets have been associated to an increased risk of periodontitis recurrence [32] and also to periodontal surgery needs [33]. A recent study has shown that PDT reduced residual pockets with PPD > 5 mm, but this reduction was mainly observed in initially deep and bleeding upon probing periodontal sites, highlighting the influence of local factors on PDT effectiveness [29].

The aim of this study was to present an additional analysis of data from a split-mouth double-blind randomized clinical trial [29], comparing the effect of repeated PDT applications at molar furcation sites versus other sites of molars and non-molars during non-surgical active therapy of severe periodontitis at 3- and 6-months.

## 2. Materials and Methods

### 2.1. Study Population and Inclusion/Exclusion Criteria

This is an additional analysis of a study approved by the local Institutional Ethical Committee (University Hospitals of Strasbourg, ClinicalTrials.gov Identifier: NCT02030470) and performed according to the Declaration of Helsinki (2008). The protocol has been described in details in a previous publication [29], thus only a brief description is provided here. All participants were given written information and their written informed consent was obtained. Recruitment of patients suffering from severe generalized chronic periodontitis [34] extended from June 2014 to June 2017 at the Department of Periodontology, University Hospitals of Strasbourg, France. Demographic data, medical and dental history, and smoking status were recorded. The inclusion criteria included:

(a) at least 40 years-old;
(b) at least 20 teeth (third molars not included);
(c) at least 30% of sites with CAL > 5 mm and ≥5 sites with PPD ≥ 5 mm for each quadrant;
(d) at least one molar per quadrant (third molars not included);
(e) bone loss;
(f) bleeding on probing (BOP) ≥ 30%.

The exclusion criteria included:

(a) aggressive periodontitis [34];
(b) smokers with more than 10 cigarettes/day;
(c) antibiotic and anti-inflammatory treatments in the last six months;
(d) previous periodontal therapy;
(e) medical history likely to affect periodontal status and/or to compromise treatment outcomes;
(f) pregnant/breastfeeding patients.

### 2.2. Clinical Measurements

Clinical parameters were recorded at baseline, and again at 3- and 6-months, at six sites per tooth. Clinical parameters measurements included plaque index (PI) [35], BOP, PPD, gingival recession, and CAL measured with a PCPUNC 15 periodontal probe (Hu-Friedy, Chicago, IL, USA). Molar furcation sites included, midbuccal, and midlingual sites for mandible molars, midbuccal, mesio-palatal and disto-palatal sites for maxilla molars. The horizontal involvement of the furcation sites was assessed using a curved Nabers furcation probe PQ2N7 (Hu-Friedy, Chicago, IL, USA), and scored according to the classification of Hamp et al. [36] as follow: class 0, furcation not probable; class I, horizontal loss of periodontal tissue support < 3 mm; class II, horizontal loss of periodontal tissue support > 3 mm but not encompassing the total width of the furcation area; and class III, horizontal “through-and-through” destruction of the periodontal tissue.

The changes of pockets with PPD > 5 mm was the primary outcome. The changes of BOP and PI percentages, mean PPD, and CAL were the secondary outcomes.

### 2.3. Randomization

A randomized split-mouth double-blind controlled design (RCT) was set up as previously described [29]. Investigators (L.H., A.C., A.M., C.P., and J.-L.D.) were trained periodontists. They were not aware of treatment allocation when they performed examination and SRP, at different times. At baseline (V1), each of the four quadrants per patients was assigned to either a test group (SRP + PDT) or control group (SRP) using a randomization table with a 1:1 allocation. The same procedure was repeated with a second investigator and a third investigator, at 3-months (V2) and at 6-months (V3) respectively. The patients were blinded to the quadrants receiving PDT treatment.

### 2.4. Study Design and Treatments

At the first visit, patients received oral hygiene instructions (OHI). At V1, SRP was performed under local anesthesia at sites with PPD >3 mm [37]. SRP and PDT were performed in test quadrants [24]. Initial treatment was performed within three weeks. Patients were instructed to use a chlorhexidine mouthwash (0.12%) twice a day for 15 days. At V2, after periodontal reevaluation, SRP and PDT were carried out in residual sites with PPD >3 mm following the same quadrant allocation determined at V1.

FotoSan^®^ system (CMS Dental, Copenhagen, Denmark) was used for PDT applications [38]. It consists of a light-emitting diode (LED) with a red spectrum (wavelength: 625–635 nm) used with a photoactivatable agent toluidine blue O (TBO) at the concentration of 0.1 mg/mL (FotoSan^®^ agent medium viscosity). TBO was placed at test sites with a needle for 1 min and then irradiated by the LED for 10–30 s depending on the pocket depth with a long specific pocket tip. This first irradiation was followed by a 10 s irradiation with a blunt trans-gingival tip. Two PDT applications with an interval of one week were done at test sites at V1, and one PDT application at V2. A sham irradiation was carried out in control quadrants.

### 2.5. Examiner Calibration

Inter-examiner calibration was performed on patients not included in the study. The percentage of agreement within ±1 mm had to be at least 80%. The intra-class correlation coefficients were superior to 0.8.

### 2.6. Calculation of Sample Size

The sample size was previously estimated [29]. Twenty-eight patients were needed based on an average number of 150 sites (including 20 molar furcation sites) per patient. Thirty-six patients were included considering 20% of potential missing data.

### 2.7. Statistical Analyses

The qualitative variables and quantitative variables are described using effectives (percentages) and the mean ± standard deviation (SD) respectively. A multilevel regression model including nested random effects (sites/teeth/jaws, and subject effects) was used to compare the baseline characteristics. For quantitative variable and binomial distribution and for qualitative variable, Gamma distribution was used. Classes 0 and I and classes II and III were merged into two groups: Classes 0–I and Classes II–III. The comparison of the principal endpoint (PPD > 5 mm) was performed using multilevel logistic regression model and included a triple interaction between time (baseline, 3-months, and 6-months), treatment group (SRP or SRP + PDT) and the type of site (molar furcation sites or other sites of molars and non-molars). The treatment effect was assessed using the interaction term between time and group for other sites and the addition of the interaction term between time and group and the triple interaction term for molar furcation sites. The use of interaction terms of the mixed model makes it possible to consider potential differences for the endpoint at baseline. Odds ratios with their 95% confidence intervals were used to present the results. A *p*-value < 0.05 was considered as statistically significant. R software version 3.6.0 (2019). R Core Team was used to perform analyses. R is a language and environment for statistical computing (R Foundation for Statistical Computing, Vienna, Austria. URL: https://www.R-project.org/).

## 3. Results

### 3.1. Characteristics of Studied Population

Thirty-six patients were initially included in the study corresponding to 2814 analyzed sites in the test group (including 328 molar furcation sites and 2486 other sites) and 2802 analyzed sites in the control group (including 325 molar furcation sites and 2477 other sites). Demographic characteristics are described in Table 1. In smokers, the mean consumption was 6.5 cigarettes per day. During follow-up, eight patients were excluded. At 3-months, three patients were excluded due to the administration of antibiotics (two following an extraction and one for an endodontic abscess). At 6-months, five other patients were excluded. One moved out, one received antibiotics for medical reasons, and three did not attend the visit. No adverse effects after therapies was reported.

### 3.2. Initial Periodontal Parameters and Treatment Outcomes at Molar Furcation Sites Versus Other Sites in Test (SRP + PDT) and Control (SRP) Groups

At baseline, the mean number of teeth and the percentage of molars was 13.0 ± 1.0 and 27.7% in SRP, and 13.1 ± 1.2 and 27.8% in SRP + PDT groups. At baseline, no significant difference between treatment subgroups was observed for periodontal pockets with PPD > 5 mm, BOP %, mean PPD and CAL, and PI (Table 2). In molars, the percentages of pockets with PPD > 5 mm of non-furcation sites was also similar, 165 (36.5%) and 171(37.58%) in the SRP + PDT and SRP groups, respectively. The numbers of classes 0–I and classes II–III were similar in both treatment groups. The percentage of pockets with PPD > 5 mm in classes 0–I was significantly higher (*p* = 0.023) in SRP group compared to SRP + PDT group but was similar for classes II–III in both treatment groups (Table 3).

**Figure 1:**
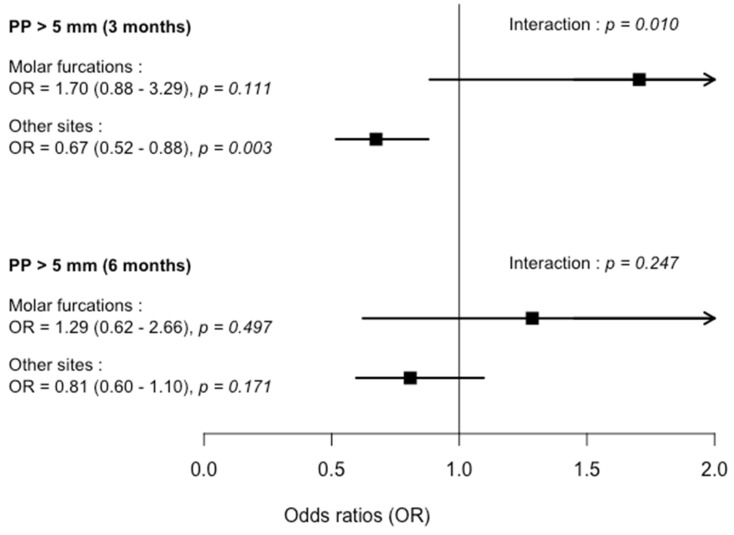
Comparison of OR. Horizontal lines represent 95% of confidence interval. PP, periodontal pockets.

A significant improvement of all clinical parameters was observed in all subgroups at 3- and 6-months compared to baseline except for PI in the molar furcations/SRP + PDT sub-group. However, the kinetics of clinical parameter changes were more dependent on site and treatment sub-groups. Between 3- and 6-months, a continuous reduction of pockets with PPD > 5 mm was observed in different sub-groups but not in the molar furcations/SRP sub-group. No additional improvement of BOP was noticed between 3- and 6-months. Conversely, an increase of BOP was observed in the molar furcations/SRP + PDT sub-group (Table 2). At 3-months, in the SRP + PDT group, a higher reduction of pockets with PPD > 5 mm (OR = 0.67, *p* < 0.003) was observed in other sites but not in molar furcation sites. A similar trend was observed for mean PPD. At 6-months, significant differences were not observed between SRP + PDT and SRP groups for all parameters (Table 4 and Figure 1). Considering non-furcation sites only in molars, no significant difference of pockets with PPD > 5 mm reductions was observed between treatment groups at 3- (OR = 0.74, confidence interval (CI) = 0.462–1.2, *p =* 0.225) and 6-months (OR = 0.79, CI = 0.473–1.34, *p =* 0.393).

## 4. Discussion

The results of this study confirmed that PDT effectiveness on residual periodontal pockets with PPD > 5 mm reduction was influenced by local risk factors such as furcation involvement. Numerous studies on additional effects of PDT have been performed and their results may appear contrasting regarding recent meta-analysis and review reports [18,19,39,40]. However, the impact of local factors on PDT effectiveness has been rarely investigated [14,29,41] while these factors, such as initial PPD, tooth type, dental plaque accumulation, and furcation involvement, have been shown to modify SRP effectiveness in the short-term [2,6,42]. Initial PPD, BOP, and tooth type (molar versus non-molar) have been shown to influence PDT residual pocket numbers [29]. Molar furcation involvement was also considered to negatively influence SRP outcomes [4,5,6,42]. This negative influence of furcation involvement on the reduction of pockets with PPD > 5 mm has been previously observed at 27-months [6].

Molar furcation involvement also appeared to negatively impact PDT effectiveness. Indeed, even if SRP + PDT significantly decreased the percentage of pockets > 5 mm by 33% (OR = 0.67) in comparison with SRP alone in non-furcation sites, there is no beneficial PDT effect at molar furcation sites. At 6-months, this trend persisted but was less pronounced, with a reduction of pockets with PPD > 5 mm by 20% (OR = 0.80) in other sites than molar furcation sites. This attenuation with time of PDT effect has been previously observed [29,43]. Interestingly, in other molar sites than molar furcation sites, the reduction of pockets with PPD > 5 mm appeared more marked in SRP + PDT group (OR = 0.74 and 0.79 at 3- and 6-months) while this difference did not reach significance level. For the other clinical parameter changes, there was no additional effect of PDT regardless of the type of site. A previous study has shown that PDT has no effect on mean PPD reduction and CAL in lingual and buccal class II molar furcations at 3- and 6-months [14]. Similarly, the use of local antimicrobial doxycycline after initial SRP treatment in furcation sites with PPD ≥ 4 mm [13] or PPD ≥ 5 mm [7] did not improve mean PPD reduction and CAL, as well as the number of persisting sites with PPD ≥ 4 mm [13]. However, in these studies, non-furcation sites have not been included and it is difficult to determine if this absence of local antimicrobial treatment effect was specifically due to furcation involvement.

Interestingly, from 3- to 6-months, a continuous reduction of pockets with PPD > 5 mm was observed in both treatment groups in other sites than molar furcation sites while in molar furcation sites, this reduction was only observed in PDT group. This delayed reduction of deep pockets in molar furcations has been previously observed for SRP alone [4,5] and could explain the absence of detected additional clinical improvement at 6-months with PDT. Indeed, the positive impact of PDT on periodontal pathogens and inflammatory cytokines in class II molar furcation sites at 6-months previously observed [14] as well as the PDT effect showed here in other sites than molar furcation sites suggest that PDT may potentially improve periodontal conditions in these sites. The weak PI improvement observed here in molar furcations could reduce PDT impact, as previously shown for whole sites [29]. The absence of PDT effects in molar furcation sites could be also explained by the responses of subgingival biofilms to SRP [44,45]. In deep furcation sites, access difficulties for SRP instrumentation impaired subgingival biofilm reduction and disorganization [41,45]. However, in vitro studies have shown that the antimicrobial effect of PDT could be reduced in organized biofilm structures [22]. Furthermore, a positive charged photosensitizer, such as TBO, could attach to negative bacterial walls and produce external bacterial wall damages [22]. High anaerobic conditions may limit the production of reactive oxygen radicals. These data suggested that PDT modalities should be adapted to molar furcation sites, using longer PDT application times and/or complementary potentiating agents such as potassium iodide [22]. The specific beneficial impact of more repeated PDT in molar furcation sites could not be excluded as observed during supporting periodontal therapy for residual pockets [46].

The fact that molar furcation sites did not respond to PDT compared to other sites may also be due to their clinical characteristics. The difficulty to place the photosensitizer as well as the activating light source in deep inter-radicular defect due to their horizontal and vertical components and the straight morphology of pocket tip could influence PDT efficacy [47]. The impact of horizontal/degree furcation involvement has been mainly demonstrated for tooth loss [9,10,11,12]. Deep pockets at furcation site have been shown to negatively influence periodontal treatment outcomes in the short-term [4,5,7,13]. In the present study, molar furcation classes were merged into groups according to their responses to periodontal treatment, i.e., no additional risk for classes 0 and I and additional risk for classes II and III [9,15,48]. The initial percentage of classes II–III (21.3%) was similar to percentages previously observed in other studies (19%) [13,15]. The percentage of pockets with PPD > 5 mm was twice more elevated in classes II–III than in classes 0–I. This impairment of periodontal condition has been previously observed for mean PPD [7,48] suggesting that patient profile in the present study was representative of severe periodontitis conditions. The impact of furcation involvement on complementary antimicrobial treatment effectiveness in the short-term has been mainly evaluated on furcation degree changes [7,15] while the persistence of classes II and III at the end of active periodontal therapy was strongly associated to long-term molar loss [9,10,48]. However, the vertical furcation involvement based on bone loss measurement has also been shown to notably influence molar loss [48], suggesting that the reduction of deep pocket was still a major goal of active periodontal therapy whatever furcation involvement.

This study was an additional analysis of a split-mouth trial designed to evaluate the impact of local risk factors and PDT on the percentage of residual deep pockets (PPD > 5 mm) at 3- and 6-months and therefore the associated needs of periodontal surgery. Considering their initial distribution in the different site/treatment subgroups, analysis of pocket with PPD > 5 mm changes has been privileged limiting PDT impact evaluation especially at 6-months. However, in spite of the high number of investigated sites, almost 250 molar furcation sites per treatment group, the power of multilevel analysis of the influence of furcation involvement degrees and other local risk factor was limited.

## 5. Conclusions

This study showed that PDT efficiency was significantly and negatively influenced by the presence of molar furcation involvement. In molar furcation, PDT did not improve the reduction of residual pockets with PPD > 5 mm contrary to other sites. The anatomical conditions, the biofilm/pocket ecology, as well as the difficulty to control dental plaque at these sites could explain this absence of PDT effect at 6-months.

## Figures and Tables

**Table 1:** Demographic characteristics at baseline.

	*N* = 36
Age (years)	50.25 ± 5.98
Women, *n* (%)	22 (61.1)
Smoker *n* (%)	11 (30.5)

**Table 2:** Pockets > 5 mm, BOP, mean PPD, mean CAL, and PI in molar furcation sites and other sites at baseline, as well as at 3- and 6-months in SRP + PDT and SRP groups.

	Molar Furcations		Other Sites	
	SRP + PDT	SRP	SRP + PDT	SRP
PPD > 5 mm nb (%)				
Baseline	70(21.34)	87(26.77)	509(20.47)	522(21.07)
3-months	42(14.09)	37(12.80)	165(7.31)	236(10.41)
6-months	***26(10.48)***	29(11.33)	**115(6.01)**	**142(7.39)**
BOP nb (%)				
Baseline	215(64.76)	207(64.89)	1597(64.60)	1615(64.91)
3-months	111(37.50)	128(43.84)	715(31.72)	764(33.78)
6-months	***107(43.32)***	107(41.80)	594(31.08)	616(31.97)
Mean PPD mm (SD)				
Baseline	4.28(1.77)	4.32(1.72)	4.02(1.70)	4.06(1.71)
3-months	3.68(1.84)	3.71(1.61)	3.12(1.54)	3.23(1.58)
6-months	**3.37(1.57)**	**3.44(1.51)**	**2.87(1.40)**	**2.87(1.38)**
Mean CAL mm (SD)				
Baseline	5.18(2.14)	5.18(2.16)	4.73(2.05)	4.71(2.04)
3-months	4.68(2.30)	4.73(2.12)	4.03(1.97)	4.06(1.97)
6-months	4.52(2.18)	4.60(2.09)	**3.86(1.95)**	**3.82(1.88)**
PI > 1 nb (%)				
Baseline	55(16.65)	64(19.81)	551(22.23)	557(22.37)
3-months	58(19.66)	57(19.59)	337(14.99)	308(13.67)
6-months	**29(13.28)**	**34(13.28)**	**213(11.09)**	**242(12.66)**

SRP, scaling and root planning; PDT, adjunctive photodynamic treatment; PI, plaque index; BOP, positive bleeding on probing; PPD, probing pocket depth; CAL, clinical attachment level; nb, number. Numbers: in bold represent *p* < 0.05 and in bold italic represent *p* ≤ 0.1 for significant change of clinical parameters between 3- and 6-months compared to baseline. Underlined numbers represent *p* > 0.05, i.e., no significant change of clinical parameters at 3- or 6-months compared to baseline.

**Table 3:** Characteristics of molar furcations at baseline in SRP + PDT and SRP groups.

	Molar Furcations	
	SRP + PDT	SRP
PPD > 5 mm nb (%)	70(21.3)	87(26.8)
PPD > 6 mm nb (%)	34(10.4)	38(11.7)
Cl 0–I nb (%)	186(81.2)	176(76.7)
Cl II–III nb (%)	43(18.7)	55(23.8)
PPD > 5 mm Cl 0–I nb (%)	30(16.1)	43(24.4)
PPD > 5 mm Cl II–III nb (%)	17(39.9)	24(43.6)

SRP, scaling and root planning; PDT, adjunctive photodynamic treatment; PPD, probing pocket depth; Cl, furcation classes; nb, number.

**Table 4:** Multilevel logistic regression of PDT effect on Pockets > 5 mm, BOP, mean PPD, mean CAL, and PI changes in molar furcation sites and other sites at 3- and 6- months.

	Molar Furcations			Other Sites		
	OR/MR	CI	*p*-value	OR/MR	CI	*p*-value
PPD > 5 mm nb (%)						
3-months	1.70	(0.884–3.289)	0.111	0.67	(0.517–0.879)	0.003 *
6-months	1.28	(0.622–2.658)	0.497	0.80	(0.596–1.096)	0.171
BOP nb (%)						
3-months	0.70	(0.418–1.190)	0.191	0.87	(0.722–1.053)	0.154
6-months	1.09	(0.638–1.877)	0.744	0.91	(0.753–1.117)	0.391
Mean PPD mm (SD)						
3-months	1.010	(0.933–1.094)	0.800	0.975	(0.948–1.004)	0.089
6-months	0.993	(0.914–1.079)	0.871	1.006	(0.976–1.036)	0.713
Mean CAL mm (SD)						
3-months	0.996	(0.912–1.088)	0.929	0.984	(0.954–1.016)	0.331
6-months	0.984	(0.898–1.078)	0.732	1.006	(0.973–1.040)	0.733
PI > 1 nb (%)						
3-months	1.38	(0.729–2.632)	0.320	1.15	(0.908–1.476)	0.237
6-months	1.09	(0.530–2.243)	0.813	1.15	(0.887–1.500)	0.287

SRP, scaling and root planing; PDT, adjunctive photodynamic treatment; PI, plaque index; BOP, positive bleeding on probing; PPD, probing pocket depth; CAL, clinical attachment level; nb, number; MR, mean ratio; OR, odds ratio; CI, confidence interval; SD, standard deviation; * significant (*p* < 0.05) difference between SRP + PDT and SRP groups.
